# Human plasma dynamically quenches the fluorescein at the initial point of oxygen radical absorption capacity (ORAC) assay

**DOI:** 10.1186/s13104-019-4845-4

**Published:** 2019-12-16

**Authors:** Harshi Gunawardena, Renuka Silva, Pathmasiri Ranasinghe

**Affiliations:** 1 Department of Applied Nutrition, Faculty of Livestock, Fisheries & Nutrition, Wayamba University of Sri Lanka, Makandura, Gonawila (NWP), Sri Lanka; 2 Herbal Technology Division, Industrial Technology Institute, Malabe, Sri Lanka

**Keywords:** ORAC assay, Fluorescence, Fluorescein, Quenching, Plasma biomolecule

## Abstract

**Objective:**

Oxygen radical absorbance capacity (ORAC) assay measures the quenching of fluorescent probe by peroxyl radicals. Antioxidants present in biological systems block the quenching of fluorescence probe. We experienced the dynamic quenching of fluorescein, the fluorescence probe used in ORAC assay by the human plasma while plasma ORAC assay was optimized. Therefore, for the first time, we report the quenching of fluorescein by human plasma at the initial point of ORAC assay.

**Results:**

Aqueous whole and non-protein fractions of plasma were used in the analysis. Since the both fractions showed a similar pattern of quenching at the initial stage, quenched percentage of fluorescein was calculated and added to each sample in subsequent analysis. Addition of extra 20% fluorescein allowed plasma samples to quench the required amount of fluorescein and follow the normal decay curves afterwards. Further, change of fluorescein quenching (ΔF/F_0_) disclosed a dose dependent linear relationship with plasma (R^2^ = 0.8). It can be speculated that dynamic quenching exhibited by human plasma biomolecule/s at the initial stage would be of non-protein aqueous phase molecule/s. We suggest initiating further studies to detect, identify and quantify the fluorescein quenching biomolecules present in human plasma for further improvements in plasma ORAC assay.

## Introduction

The oxygen radical absorbance capacity (ORAC) assay is a widely accepted method of measuring the antioxidant capacity of different biological samples. It is based upon the early work of Ghiselli et al. [1] and Glazer [2], and developed further by Cao et al. [3]. ORAC measures inhibition of peroxyl radical induced oxidation by the antioxidants and therefore, reflects classical radical chain breaking antioxidant activity by H atom transfer [4]. Peroxyl radicals are formed by a free radical initiator. Peroxyl radicals subsequently react with a fluorescent probe such as fluorescein to form a non-fluorescent product, which can be quantified easily by fluorescence. In this assay, both the inhibition time and the inhibition percentage of free radical damage are taken into a single value [5]. Antioxidants present in experimental sample can delay the oxidation of fluorescent probe by the peroxyl radicals until their antioxidant activity get depleted. Therefore, the area under the curve (AUC) of the fluorescence decay curve is used to quantify the total antioxidant activity against the peroxyl radial in the sample compared to the standard curve of water soluble vitamin E analog Trolox.

Interestingly, we found that the human plasma samples initially tend to quench the fluorescence exerted by fluorescein when we tried to optimize the plasma ORAC assay. After the dynamic quenching of fluorescein at initial point, plasma samples followed normal fluorescence decay curves. Fluorescein quenching of human plasma was appeared to be quite significant. This has not been specifically studied or previously reported in literature. Therefore, the present study was conducted to assess the initial dynamic fluorescence quenching exerted by human plasma.

## Main text

### Materials and methods

#### Subjects and methods

Seventy-six (*n *= 76) type 2 diabetic patients and eighty (*n *= 80) normoglycaemic healthy volunteers were recruited to this cross sectional study. Fasting venous blood samples were collected using lithium heparin as the anticoagulant. Plasma separation was carried out in a centrifuge ((TOMOS 3-18R High speed refrigerated centrifuge, Tomos Life Science Group, USA) set at 4000 rpm for 20 min. Plasma aliquots were stored at − 80 °C until the analysis.

#### Plasma ORAC assay

Plasma aqueous phase ORAC assay was determined based on the original method of Cao and Prior in 1999 [6] with the modifications [4]. ORAC assay was conducted at 37 °C using fluorescein as the substrate and AAPH (2,2-azobis (2-amidinopropane) dihydrochloride (Sigma Aldrich, Germany) as the oxidant generator. The assay buffer, Phosphate Buffered Saline (PBS) was used as the blank. Following the centrifugation, plasma supernatant was diluted with phosphate buffered saline (PBS) in 1:80 ratio. Ten micro liters (10 µL) of the standard or diluted samples in triplicate, 100 µL of fluorescein and 40 µL of 75 mM PBS were mixed in 96 well black plates and mixed well. Then the reaction mixtures were incubated at 37 °C in Micro-plate Reader (Spectra Max Gemini, Germany) for 30 min prior to addition of 50 µL of AAPH. After achieving the complete mixing, plate was placed in the Fluorescence micro plate reader and kinetic reads were performed at 60 s intervals for 35 cycles at excitation wavelength of 494 nm and an emission wavelength of 535 nm. Fluorescein decay curves were based on individual well fluorescence relative to the initial value and derived net area under the each curve (NAUC) at 1 min intervals. NAUC of standards were used to derive the standard curve. Data were expressed as antioxidant activity in Trolox equivalents based on above derived standard curve. One ORAC unit was defined as the net protection area provided by 1 µM final concentration of Trolox.

### Results

#### Dynamic fluorescein quenching by plasma

**Fig. 1:**
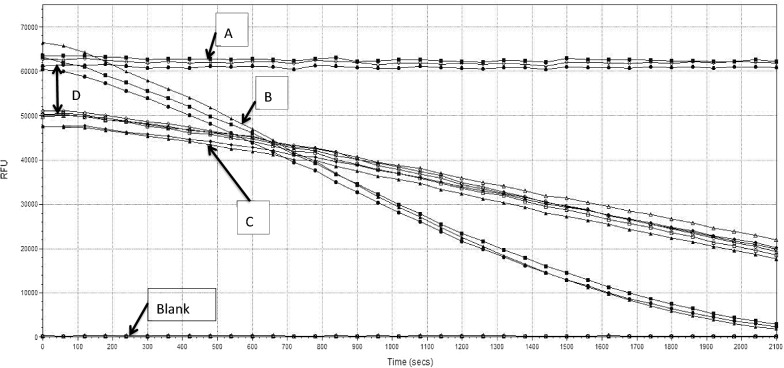
Dynamic fluorescence quenching of plasma of type 2 diabetic patients and healthy controls (Segment A—fluorescein solution in the absence of AAPH; Segment B—fluorescence decay curves of fluorescein in the absence of plasma; Segment C—fluorescence decay curves of fluorescein in the presence of human plasma; D—dynamic quenching of fluorescein solution by the plasma of both diabetic and healthy subjects at the initial point)

In the plasma ORAC assay, steady quenching of fluorescence exhibited by fluorescein over the period of time (35 cycles) monitored at 1 min intervals. However, plasma of both diabetic and normoglycaemic healthy individuals tends to quench a significant amount of fluorescein dynamically at the initial stage. Therefore, it could suspect the presence of a compound or biomolecule of plasma with the potential of dynamic quenching of fluorescence exerted by fluorescein as illustrated in Fig. 1. Further, the potent compound or biomolecule let the decay curves to be followed afterwards (Fig. 1). It was indicated in the static quenching of fluorescence over the rest of 35 cycles of the assay.

With the limited laboratory facilities, we were unable to detect the potent compound or biomolecule present in plasma with dynamic quenching properties. However, there are no any studies or published data on human serum/plasma albumin or any other protein or biomolecule present in human plasma on quenching the fluorescence emitted by fluorescein. Nevertheless, to avoid any possible interference exerted by the plasma proteins, non-protein fraction of the plasma was extracted using perchloric acid (plasma:perchloric acid, 1:1 ratio) and used in the subsequent analysis. However, fluorescence quenching of plasma non-protein fraction was similar to that of the plasma fraction with proteins. Hence, we can assume that the quenching is not exerted fully or partly by proteins present human plasma.

**Fig. 2:**
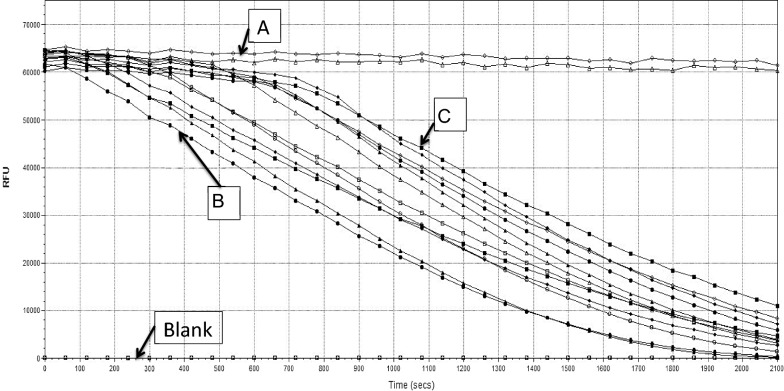
Fluorescence decay curves of plasma of diabetic patients and healthy subjects after correcting for dynamic quenching by adding extra 20 μL of fluorescein (Segment A—fluorescein solution in the absence of AAPH; Segment B—fluorscence of fluorescein solution in the absence of plasma; Segment C—fluorescence of fluorescein solution in the presence of human plasma + addition of 20 μL of extra fluorescein)

As illustrated in Fig. 1, it has observed that the quenching was stable after the initial point of ORAC assay (t = 0 s) and towards the rest of the 35 cycles (t = 2100 s) of ORAC assay over the time. Meanwhile, the initial quenching was dynamic and the change in quenching over the time (35 min) was static, percentage of fluorescence quenched by plasma samples were calculated and added that amount of fluorescein to the each well (100 µL + % fluorescein quenched). Calculated percentage of fluorescein to be added to the initial volume was 16–20%. Addition of 120 µL of fluorescein gave required initial relative fluorescence units (RFU) allowing 20% to be quenched and follow the normal fluorescence decay curves as shown in Fig. 2. Therefore, addition of 20 µL was continued in the analysis.

**Fig. 3:**
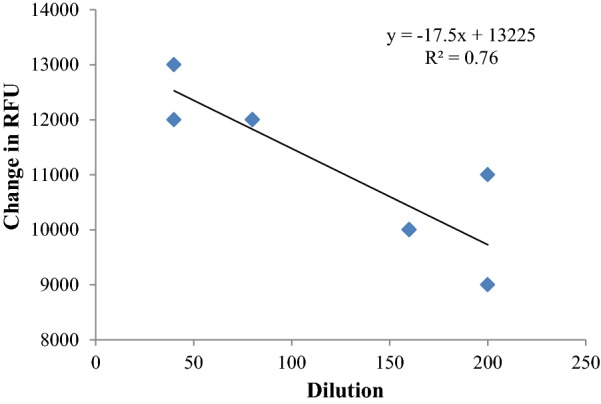
Dose dependent relationship exhibited by the different dilutions of human plasma with fluorescence decay

Dose dependency and linearity characteristics of dynamic plasma quenching were further studied using a dilution series. Dilution series of plasma as 10, 20, 40, 80, 160 and 200 were used to assess the dose dependency and it is illustrated in Fig. 3. After calculating the change of relative fluorescence units (RFU) at t = 0 of each of dilution series, it was plotted against the dilution to check the linearity of quenching and it was 0.76. Change in RFU with dilution series resembles that the quenching of fluorescence by the plasma molecule was dependent on its concentration. Hence it suggests the presence of significant linear relationship between the concentration of plasma quencher with the fluorescence quenching.

### Discussion

In the ORAC assay, oxidative degradation or quenching of fluorescence probe by the peroxyl radicals is assessed. Antioxidants can delay the quenching of fluorescence probe by peroxyl radicals. Therefore, time taken for quenching the fluorescent probe may vary depending on the antioxidant potential of the sample. Hence, in this assay, both the inhibition time and the inhibition percentage of free radical damage are taken into a single value [5]. Fluorescence quenching detects as the decline in fluorescence intensity of sample. A variety of molecular interactions can result in quenching such as excited-state reactions, molecular rearrangements, energy transfer, ground state complex formation and collisional quenching.

In fact the fluorescence quenching is used in the ORAC assay, this is the first time we report the dynamic quenching of fluorescence given by fluorescein in plasma analysis. Similar pattern of dynamic quenching exhibited for plasma of both diabetic and healthy individuals indicates the presence of a common biomolecule or compound. Although the evidence on fluorescence quenching by plasma proteins is not available, frequent use of quenching process in examining,the structural and functional properties of proteins has been reported previously [7, 8]. In eliminating any possible interference by plasma proteins, steps were taken to analyze the non-protein fraction of plasma. Aqueous non-protein fraction of the plasma was extracted using equal volumes of plasma and perchloric acid. However, our findings have shown that the non-protein fraction of plasma followed the same pattern of quenching as whole plasma. Therefore, we can infer that the quenching is not partly or fully imparted by plasma proteins. Other potential fluorescence quenchers present in human plasma are found to be molecular oxygen [9–14] and uric acid derivatives like porphyrin uric acid [15]. However, we could not recognize the molecule due to lack of facilities.

According to our findings, addition of extra fluorescein has shown to be effective in addressing the dynamic quenching by plasma. Hence, it was further confirmed that compound or biomolecule present in plasma quenches exact percentage of fluorescence provided by extra fluorescein. Plasma dilution series exhibited that the fluorescence quenching was dose dependent.

Although we were unable to identify the molecule present in the plasma with fluorescence quenching activity, we could suspect it as a molecule common for both diabetics and healthy people. Since the non-protein fraction followed the same pattern of quenching as whole plasma, it is confirmed that the quenching is not partly or fully imparted by plasma proteins. As the lipid phase extraction was not carried out in ORAC analysis, it can be predicted that the molecule or the compound of interested would be a water soluble one. It followed the dynamic florescence quenching at the initial point and exhibited static fluorescence quenching in rest of the assay. Therefore, further analysis and study of the compound or biomolecule present in the human plasma responsible for quenching would be of great importance.

### Conclusion

Fluorescence quenching by human plasma in ORAC assay was observed for the first time. Both diabetic and healthy individuals tend to have the similar pattern of fluorescence quenching. Dynamic fluorescence quenching taken place at the aqueous phase ORAC assay speculate that the interested quenching compound or biomolecule would be of water soluble nature. A significant linear relationship between the fluorescence quenching and concentration of the plasma quencher was observed. However, further analysis of fluorescence quenching by the interested molecule would be of great importance in improving the plasma ORAC assay.

## Limitations

We were not able to identify the interested biomolecule or compound responsible for dynamic quenching of fluorescein at the initial point of plasma ORAC assay due to unavailability of laboratory facilities.

In fact, we could not able to identify and quantify the biomolecule or compound responsible for dynamic quenching, only the possible interference was minimized by adding extra fluorescein.

Only we could infer the nature of interested biomolecule or compound as it is a water soluble and common molecule for both diabetic and healthy individuals.

Although the non-protein fraction demonstrated the similar pattern of dynamic quenching as whole plasma, contribution of protein derivatives might have interfered.

## Data Availability

The datasets analysed during the current study are available from the corresponding author on reasonable request.

## Abbreviations

AUC: area under the curve
NAUC: net area under the curve
ORAC: oxygen radical absorption capacity
RFU: relative fluorescence units
